# Overexpression of Heme Oxygenase-1 Prevents Renal Interstitial Inflammation and Fibrosis Induced by Unilateral Ureter Obstruction

**DOI:** 10.1371/journal.pone.0147084

**Published:** 2016-01-14

**Authors:** Xiao Chen, Shi-Yao Wei, Jian-Si Li, Qing-Fang Zhang, Yu-Xiao Wang, Shi-Lei Zhao, Jing Yu, Chang Wang, Ying Qin, Qiu-Ju Wei, Gui-Xiang Lv, Bing Li

**Affiliations:** 1 Department of Nephrology, 2nd Affiliated Hospital of Harbin Medical University, Harbin, China; 2 Department of Biochemistry and Molecular Biology, Harbin Medical University, Harbin, China; INSERM, FRANCE

## Abstract

Renal fibrosis plays an important role in the onset and progression of chronic kidney diseases. Many studies have demonstrated that heme oxygenase-1 (HO-1) is involved in diverse biological processes as a cytoprotective molecule, including anti-inflammatory, anti-oxidant, anti-apoptotic, antiproliferative, and immunomodulatory effects. However, the mechanisms of HO-1 prevention in renal interstitial fibrosis remain unknown. In this study, HO-1 transgenic (TG) mice were employed to investigate the effect of HO-1 on renal fibrosis using a unilateral ureter obstruction (UUO) model and to explore the potential mechanisms. We found that HO-1 was adaptively upregulated in kidneys of both TG and wild type (WT) mice after UUO. The levels of HO-1 mRNA and protein were increased in TG mice compared with WT mice under normal conditions. HO-1 expression was further enhanced after UUO and remained high during the entire experimental process. Renal interstitial fibrosis in the TG group was significantly attenuated compared with that in the WT group after UUO. Moreover, overexpression of HO-1 inhibited the loss of peritubular capillaries. In addition, UUO-induced activation and proliferation of myofibroblasts were suppressed by HO-1 overexpression. Furthermore, HO-1 restrained tubulointerstitial infiltration of macrophages and regulated the secretion of inflammatory cytokines in UUO mice. We also found that high expression of HO-1 inhibited reactivation of Wnt/β-catenin signaling, which could play a crucial role in attenuating renal fibrosis. In conclusion, these data suggest that HO-1 prevents renal tubulointerstitial fibrosis possibly by regulating the inflammatory response and Wnt/β-catenin signaling. This study provides evidence that augmentation of HO-1 levels may be a therapeutic strategy against renal interstitial fibrosis.

## Introduction

Progressive renal tubulointerstitial fibrosis is a common pathway leading to end-stage renal diseases.[1–3] Tubulointerstitial fibrosis is characterized by the destruction of renal tubules, infiltration of inflammatory cells, rarefaction of the peritubular microvasculature, accumulation of myofibroblasts, and excessive deposition of extracellular matrix (ECM), which are regulated by many signaling pathways and thought to play pivotal roles in the pathogenesis of chronic kidney diseases (CKDs).[1–3] Therefore, it is important to prevent renal interstitial fibrosis to slow the progression of CKD. However, effective therapeutic strategies are still limited.

Heme oxygenase-1 (HO-1) converts heme to biliverdin via a reaction that produces carbon monoxide and liberates iron. Researchers have demonstrated that HO-1 is involved in anti-inflammatory, anti-oxidant, anti-apoptotic, antiproliferative and immunomodulatory effects that protect diverse organs against injury, including acute kidney injury (AKI).[4] Nevertheless, even though the salutary effects of HO-1 during short-term renal stress have been established, it is not clear whether such a paradigm can be extended to chronic renal fibrosis. Previous studies reported that HO-1 was upregulated in tubular epithelial cells of the human kidney in various renal diseases, epithelia are more susceptible to oxidative stress due to the lack of this critical enzyme,[5] and HO-1 deficiency promotes epithelial-mesenchymal transition and renal fibrosis.[6] However, whether overexpression of HO-1 is beneficial or detrimental in kidney disease is unclear. Myocyte-restricted HO-1 transgenic (TG) mice exhibited significantly increased post-infarction survival and decreased left ventricular dilatation, mechanical dysfunction, hypertrophy, interstitial fibrosis, and oxidative stress during chronic heart failure.[7] Although induction of HO-1 halts renal interstitial fibrosis, [8] the potential mechanisms remain unclear.

Tubular atrophy is associated with interstitial fibrosis and most likely has distinct mechanisms related to rarefaction of the peritubular microvasculature and tubular cell loss.[1, 2] The improvement of renal function mainly depends on the repair and regeneration of injured tubular epithelial cells. Sterile inflammation contributes to tubular atrophy and interstitial fibrosis,[9] causing an increase not only in the production of inflammatory cytokines but also in the infiltration of neutrophils and macrophages. Macrophages, a major type of inflammatory cell, are recruited in all forms of kidney disease that involve inflammation. Recruited macrophages are implicated in the induction of renal injury, repair, and fibrosis.[10–12] The continued presence of abnormal quantities of inflammatory macrophages leads to renal injury, and the phenotypic switch to reparative macrophages ultimately causes renal fibrosis. However, in our previous study, macrophages promoted the repair of kidney injury via the canonical Wnt signaling pathway.[13] Wnt signaling is a complex, highly conserved, cell-to-cell communication pathway in multicellular organisms that regulates cell fate, function and phenotype during development and is also often involved in diseases, including those involving the kidney.[14, 15] Evidence has shown that Wnt/β-catenin signaling promotes renal interstitial fibrosis [15] and that the delivery of a Wnt antagonist, the DKK1 gene, reduces β-catenin accumulation and attenuates renal interstitial fibrosis in a mouse UUO model.[16]

In the present study, HO-1 TG mice were used to induce a UUO model. We investigated whether increased HO-1 expression was able to ameliorate renal interstitial inflammation and fibrosis and explored the potential mechanisms.

## Materials and Methods

### Animals and animal models

Transgenic mice systemically overexpressing HO-1 (TG) were generated via pronuclear microinjection. Fertilized eggs from C57BL/6 F1 parents were injected with a transgenic construct expressing cDNA for mouse HO-1 and the Gly143 to His (G143H) mutant of HO-1 under the control of the chicken β-actin promoter.[17] Their non-transgenic male littermates (WT) were used as controls. Male TG mice overexpressing the HO-1 gene were identified by polymerase chain reaction (PCR) analysis using tail DNA. The original mouse breeding colony was obtained from the biochemical laboratory of Harbin Medical University (Harbin, China). All mice were maintained in filter-top cages and received sterilized food and acidified water. All experiments were conducted in accordance with the National Institutes of Health Guidelines for the Care and Use of Laboratory Animals. Experimental protocols were approved by the animal committee of Harbin Medical University. At 8 to 12 weeks of age, the body weight (BW) of the mice was calculated, and they underwent surgery to induce UUO. The left ureter was exposed but not ligated in the sham mice used as controls. The sham and UUO mice were divided into two groups: the TG group and the WT group. Twenty TG mice and 20 WT mice in each group were sacrificed on days 3, 7, 10, and 14 after UUO surgery (n = 5 per time point). Five TG mice and 5 WT mice were sacrificed on day 3 after the sham operation.

### Blood and tissue samples

Renal function was assessed by measuring serum creatinine levels with automatic biochemistry analyzer (Roche, Cobasc 311, Mannheim, Germany). The mice were perfused with ice-cold normal saline via the left ventricle for 2 minutes, and the kidney was rapidly excised. Tissues were also divided for histopathological analysis and molecular biological measurements, and portions of the kidney were fixed or snap-frozen in liquid nitrogen until analysis.

### Histological studies and immunostaining

As described previously,[13, 18] a subsample of each kidney tissue sample was embedded in paraffin (stored at 4–8°C), and another subsample was embedded in Tissue-Tek O.C.T. compound (Sakura Finetek, Torrance, CA) (stored at -80°C). Frozen sections were obtained using a cryostat (Thermo Scientific, Cheshire, UK) at 4 μm, and paraffin sections were obtained using a microtome (Thermo Scientific, Walldorf, Germany) at 2–3 μm. Paraffin sections were stained with hematoxylin-eosin (HE), Masson’s trichrome, and picrosirius red using standard techniques. TGF-β1 and Ki-67 staining of paraffin sections was performed by dewaxing, antigen retrieval (citric acid buffer incubation and microwave heating), and antibody incubation. Additional immunolabeling was performed on frozen sections. Immunofluorescence labeling was performed using a previously described protocol.[19] Primary antibodies against the following proteins were used for immunolabeling: rabbit anti-mouse HO-1 (1:200), rabbit anti-mouse TGF-β1 (1:100, Abcam, Hong Kong), rat anti-mouse F4/80 (1:200, clone BM8; eBioscience, San Diego, CA), rat anti-mouse E-cadherin (1:200; Abcam, Hong Kong) anti-mouse α-SMA-Cy3 (1:200, mouse Cy3-conjugated clone 1A4; Sigma, St. Louis, MO), rat anti-mouse PDGFRβ (1:200, clone APB5; eBioscience), rabbit anti-mouse Ki-67 (1:200, clone SP6; Thermo Scientific, Fremont, CA), rabbit anti-mouse CD31 (1:50, Abcam, Hong Kong), and rabbit anti-mouse β-catenin (1:200; Abcam, Hong Kong). The secondary antibodies were Alexa Fluor 594-conjugated goat anti-rabbit, Alexa Fluor 488-conjugated goat anti-rabbit, and Alexa Fluor 488-conjugated goat anti-rat antibodies (1:200; Jackson ImmunoResearch Laboratories, West Grove, PA). Nuclei were stained using 4, 6-diamidino-2-phenylindole (DAPI). The secondary antibody of immunohistochemistry was horseradish peroxidase-conjugated goat anti-rabbit IgG (BOSTER, Wuhan, China), and coloration was performed with a DAB Kit (BOSTER, Wuhan, China). Cell nuclei were stained with hematoxylin. Apoptotic cells were detected using a TUNEL Apoptosis Assay Kit (Roche, USA). Images were captured using a Nikon microscope (Tokyo, Japan) and processed using NIS-Elements software (Tokyo, Japan).

All tissue section analyses were performed using 10–15 random cortical interstitial fields per mouse. The data obtained from each tissue are represented by the mean of all fields. The degree of tubulointerstitial injury was determined in HE-stained paraffin sections as previously described.[20] Interstitial fibrosis was quantified as the picrosirius red-positive area and the blue area of Masson-stained sections. The expressions TGF-β1 were graded on a scale from 0–4 as follows: grade 0, normal; grade 1, mild staining; grade 2, moderate staining; grade 3, strong staining; and grade 4, extremely strong staining. F4/80-positive cells; Ki-67-positive cells; and PDGFRβ-, α-SMA-, HO-1 and β-catenin-stained areas were quantified as previously described.[20] CD31-labeled kidney sections were used to evaluate PTC loss as follows: each image was divided into 252 squares, one square without a PTC was considered positive for loss, and the final score was presented as the percentage of positive squares.

### Quantitative real-time PCR analysis

Extraction of RNA from cryopreserved mouse kidneys, cDNA synthesis, and real-time PCR were performed using previously described methods.[20] Valid primer sequences are listed in Table 1. Cycle threshold (CT) values of each mRNA were normalized to those of β-actin. Fold differences were determined using the 2^–ΔΔCT^ method and analyzed relative to those of WT mice in the sham group.

**Table 1:** Primers used for quantitative real-time PCR.

Studied Gene	Sequence of Oligonucleotides (5’-3’)	Studied Gene	Sequence of Oligonucleotides (5’-3’)
HO-1		WNT10b	
F	CAGCCCCACCAAGTTCAAAC	F	GGCTGTAACCACGACATGGA`
R	GTCTTTGTGTTCCTCCTCTGTCAGCAT	R	GCACTTCCGCTTCAGGTTTT
TGF-β1		TNF-α	
F	GCCTGAGTGGCTGTCTTTTGA	F	GCCACCACGCTCTTCTGTCT
R	TCCAACCCAGGTCCTTCCTA	R	TTGACGGCAGAGAGGAGGTT
CTGF		IL-1β	
F	TCCCGAGAAGGGTCAAGCT	F	CTTTCCCGTGGACCTTCCA
R	GACAGGCTTGGCGATTTTAGG	R	GGGTGTGCCGTCTTTCATTAC
WNT2b		IL-6	
F	CCACCCGGACTGATCTTGTC	F	GAGTTGTGCAATGGCAATTCTG
R	ACCCTCGGCCACAACACAT	R	CACTCCTTCTGTGACTCCAGCTT
WNT4		MCP-1	
F	GCTCGGACAACATCGCCTAT	F	AAAACCTGGATCGGAACCAAA
R	CCACCCGCATGTGTGTCA	R	TGCTTGAGGTGGTTGTGGAA
WNT5a		MIP-1	
F	GAAGCAGGCCGTAGGACAGT	F	TGCCTGCTGCTTCTCCTACA
R	CGCGCTATCATACTTCTCCTTGA`	R	TGGACCCAGGTCTCTTTGGA
WNT5b		IL-4	
F	GGTCGCCTCTGCAACAAGAC	F	TATCCACGGATGCGACAAAA
R	GGTGCATTTTTTGCATCTGACA	R	GCCCTACAGACGAGCTCACTCT
WNT7b		IL-10	
F	CTGCAGCCAGGGCAATCT	F	CAGCCGGGAAGACAATAACTG
R	ATCTCACGGGCATCCACAAA	R	CCACTGCCTTGCTCTTATTTTCA
WNT10a		β-actin	
F	GGCTGCAGTCCGGATGTG	F	ACGGCCAGGTCATCACTATTG
R	GCACTTACGCCGCATGTTC	R	CCTGCTTGCTGATCCACATCT

Note. F: Forward; R: Reverse

### Western blotting analysis

The preparation of kidney tissue homogenates and western blotting analysis were performed as previously described.[20] For the detection of TGF-β1 and β-catenin, blots were incubated with rabbit anti-mouse TGF-β1(1:5000; Abcam, Hong Kong) and rabbit anti-mouse β-catenin (1:10,000; Abcam, Hong Kong) followed by incubation with horseradish peroxidase-conjugated goat anti-rabbit IgG (1:10,000; Abcam, Hong Kong). All western blot results were normalized to β-actin.

### Enzyme-linked immunosorbent assay (ELISA)

Mouse TNF-α, IL-1β, IL-6, IL-4 and IL-10 ELISA kits (R&D Systems, Minneapolis, MN, USA) were used to determine the concentrations of these markers in kidney tissue homogenates. Briefly, 50μl of standard were added to the standard well, 10μl tissue homogenate and 40ul Sample Diluent was added to each sample well. For each well, horseradish peroxidase (HRP)-conjugated reagent was applied and incubated for 1 hour at 37°C. Tetramethylbenzidine (TMB) was added after washing. The chromogenic reaction was terminated with Stop Solution. Absorbance was measured at 450 nm with an iMark^™^ Microplate Reader (BIO-RAD, Japan). The BCA Protein Assay Kit was used to determine the total protein concentrations of the kidney tissue homogenates.

### Statistical analysis

All data are presented as the mean ± standard deviation (SD). Statistical analyses were conducted using one-way ANOVA with the Hochberg test and two-sample t tests. All tests were conducted using SPSS 17.0 software (SPSS Inc., Chicago, IL, USA); p<0.05 was considered statistically significant, and p<0.01 was considered highly significant.

## Results

### HO-1 is adaptively upregulated in injured kidneys following UUO, and is persistently overexpressed in HO-1 transgenic kidneys

**Fig 1:**
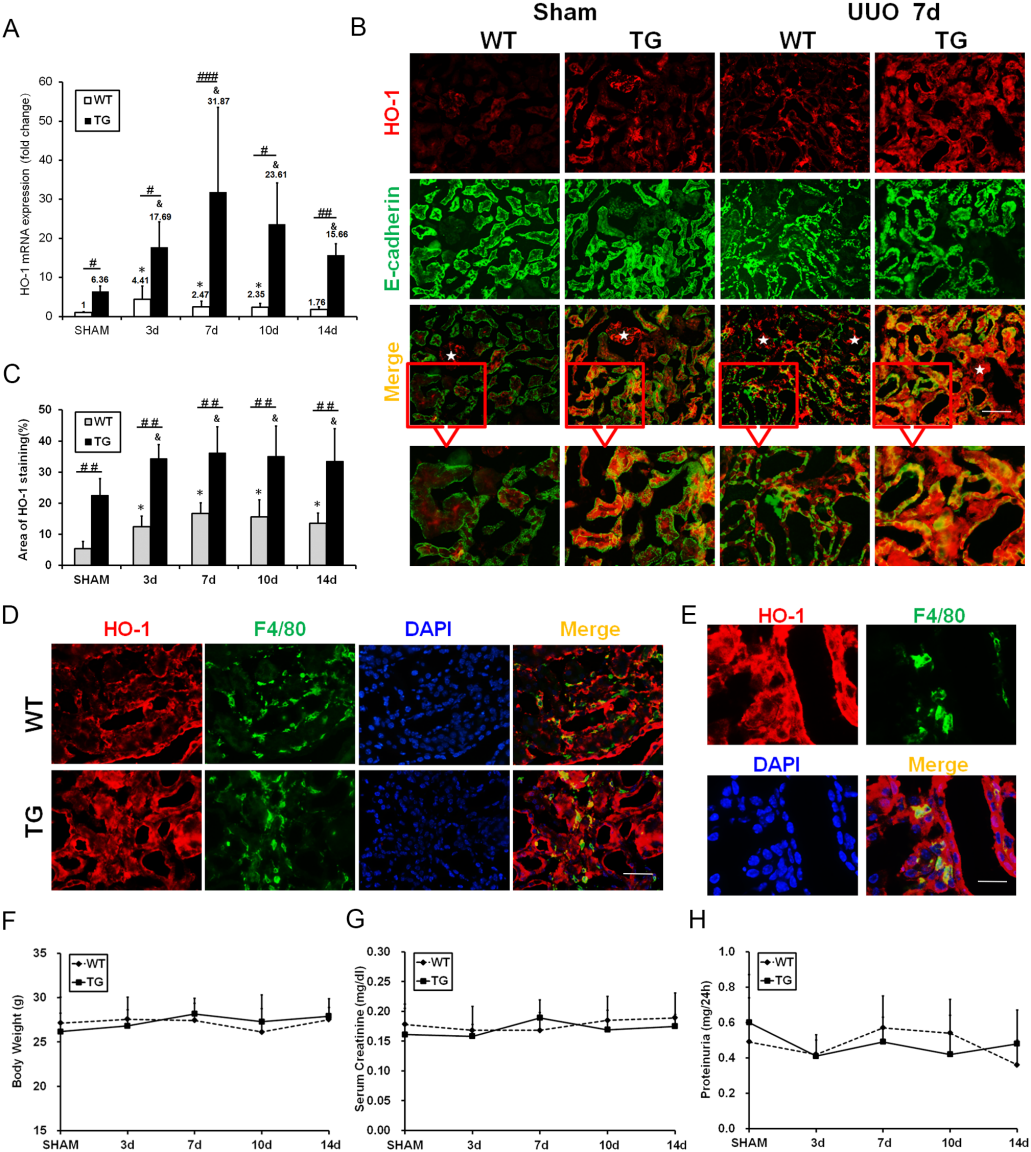
HO-1 was adaptively upregulated in injured kidneys and persistently overexpressed in transgenic kidneys. **(A)** Analysis of HO-1 mRNA levels in kidneys from TG and WT mice after UUO. **(B)** Representative immunofluorescence images of kidney sections co-labeled with HO-1 (red) and E-cadherin (green) in the Sham and UUO groups on day 7. (Magnification, ×200; bars = 250 μm) White stars indicate glomerulus. **(C)** Graph showing the area of HO-1 staining. **(D)** Representative immunofluorescence images of kidney sections co-labeled with HO-1 (red) and F4/80 (green) in the WT and TG groups at 7 day after UUO. (Magnification, ×400; bars = 125μm) (E) Representative higher magnifications views of dual immunostaining with HO-1 (red) and F4/80 (green) after UUO. (Magnification, ×1000; bars = 50 μm). (F-H) Line graphs indicating that there were no differences in body weight (F) before surgery or in serum creatinine levels (G) or proteinuria (H) after sham or UUO surgery between the WT and TG groups. * P<0.05, ** P<0.01, *** P<0.001 vs. WT mice of the Sham group; ^&^P<0.05, ^&&^P<0.01 vs. TG mice of the Sham group; #P<0.05, ## P<0.01, ### P<0.001 vs. WT mice of the respective groups.

To determine the effect of HO-1 on renal interstitial fibrosis, TG mice that express high levels of HO-1 were used to establish the UUO model. The expression of kidney HO-1 mRNA was detected. It obviously increased in the TG group compared with the WT group, especially after UUO injury, and remained high throughout the entire experimental process (Fig 1A). To investigate the origin of HO-1 in kidneys, dual immunofluorescent staining with HO-1 and E-cadherin (a marker of tubules) was performed. As shown in Fig 1B, small amount of HO-1 expression was observed in glomeruli and it was scarcely detected in tubules and interstitium from WT mice in the sham group. However, increased HO-1 expression was observed in tubules and interstitium after UUO injury in WT mice (Fig 1B). In TG mice, HO-1 overexpression could be clearly observed in tubules and interstitium under normal conditions. Furthermore, HO-1 expression was obviously enhanced in tubules and interstitium after UUO injury (Fig 1B). The index of HO-1 staining is expressed graphically in Fig 1C. We noticed that most of HO-1 positive cells co-stained with tubular epithelial cells (E-cadherin positive), suggested that tubules were the main origin of HO-1. To identify the other cellular origin of HO-1 in tubulointerstitium after UUO injury, dual immunofluorescent staining with HO-1 (red) and F4/80 (green) was performed (Fig 1D and 1E). Some HO-1- positive interstitial cells co-labeled with macrophages, indicated that part of HO-1 secreted by macrophages. There were no significant differences in weight, serum creatinine, or proteinuria between TG and WT mice (Fig 1F–1H).

### Overexpressed HO-1 reduces renal tubulointerstitial injury and fibrosis after UUO induction

**Fig 2:**
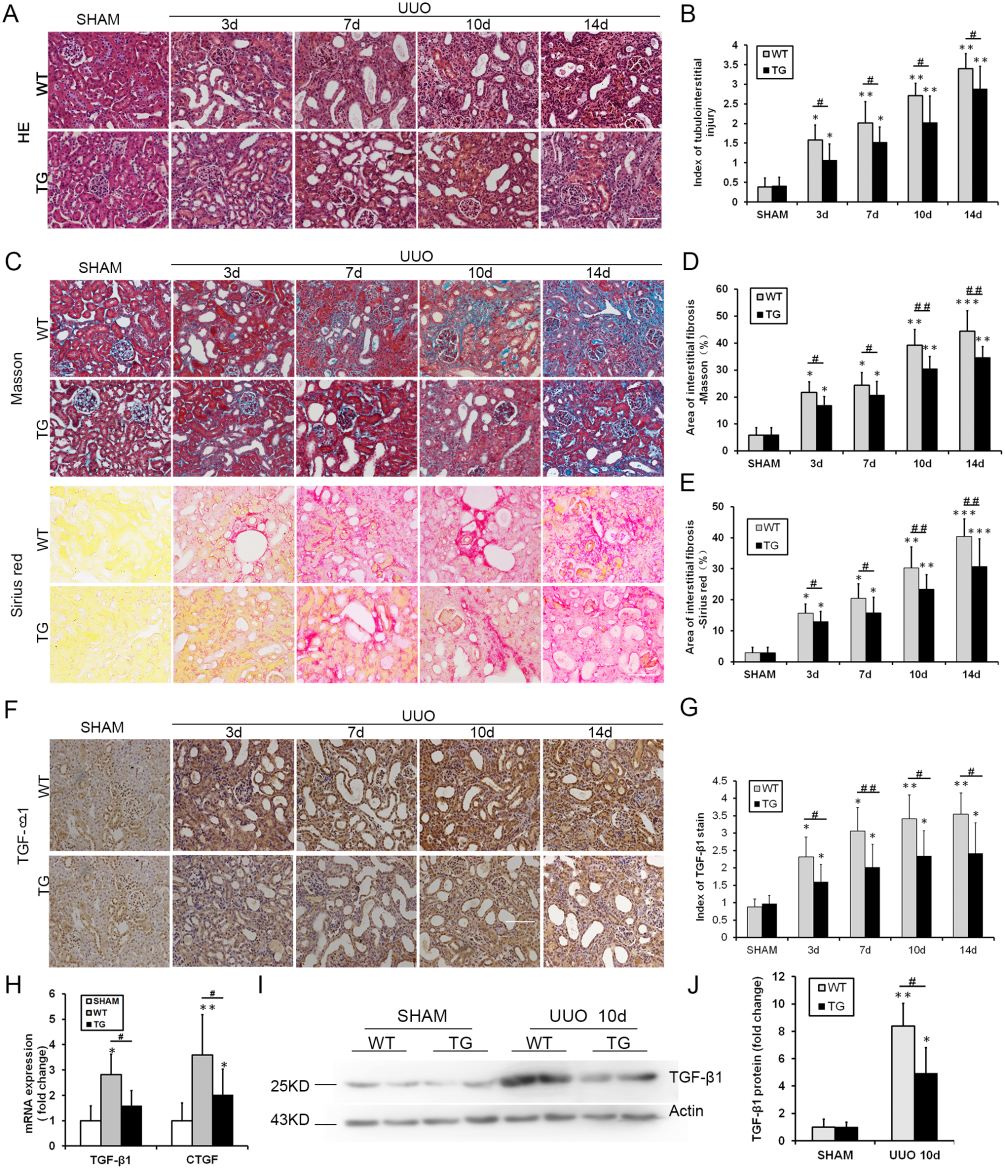
HO-1 reduced renal tubulointerstitial injury and fibrosis after UUO. **(A)** Representative light microscopy images of HE-stained kidney sections from the Sham and UUO groups on days 3, 7, 10 and 14 (magnification, ×200; bars = 250 μm). **(B)** Histogram showing the semi-quantitative determination of tubulointerstitial lesions. **(C)** Representative light microscopy images of Masson-stained and picrosirius red-stained kidney sections from the Sham and UUO groups on days 3, 7, 10 and 14 (magnification, ×200; bars = 250 μm). **(D)** Representative light microscopy images of picrosirius red-stained kidney sections from the Sham and UUO groups on days 3, 7, 10 and 14 (magnification, ×200; bars = 250 μm). **(E)** Histogram showing the area of picrosirius red-positive staining per high-power field in mice of the WT and TG groups after sham or UUO surgery. **(F)** Representative images of TGF-β1 immunohistochemical staining (brown) of kidney sections from the Sham and UUO groups at 3, 7, 10 and 14 days (magnification, ×200; bars = 250 μm). **(G)** Graph showing a semi-quantitative index of TGF-β1 staining. **(H)** Histogram showing TGF-β1 and CTGF mRNA levels at day 10 after UUO. The results are representative of triplicate analyses. **(I-J)** Representative Western blot (I) and quantitative data (J) for renal TGF-β1 expression 10 days after sham or UUO surgery. The data are expressed as the mean ± SD. * P<0.05, ** P<0.01, *** P<0.001 vs. WT mice of the Sham group; #P<0.05, ## P<0.01 vs. WT mice of the respective groups.

As anticipated, extensive fibrosis, significant tubular epithelial cell necrosis, tubular dilation, and inflammatory cell infiltration worsened over time after UUO. In contrast, these alterations were notably ameliorated in TG mice compared with WT mice at every time point following UUO surgery (Fig 2A). The same result is expressed graphically in Fig 2B. Increased renal fibrosis in UUO was confirmed by Masson’s trichrome staining and picrosirius red staining (Fig 2C). However, these changes were strikingly decreased in the TG group compared with the WT group (Fig 2C). The same result is expressed graphically in Fig 2D and 2E. These data demonstrated that the overexpression of HO-1 ameliorated tubular injury and renal interstitial fibrosis. Meanwhile, changes in fibrosis-related growth factors, namely, transforming growth factor β1 (TGF-β1) and connective tissue growth factor (CTGF), were also observed in this study. Immunohistochemical staining of TGF-β1 yielded a similar decrease in the TG group compared with the WT group after UUO (Fig 2F–2G). The expression profiles of two mRNAs were also obviously decreased in the TG group compared with the WT group on the 10^th^ day following UUO (representative profiles are shown in Fig 2H). TGF-β1 yielded a similar decrease by western blots analysis in the TG group compared with the WT group on the 10^th^ day after UUO (Fig 2I and 2J).

### HO-1 overexpression prevents peritubular capillary (PTC) loss and inhibits the proliferation and activation of renal interstitial myofibroblasts after UUO

**Fig 3:**
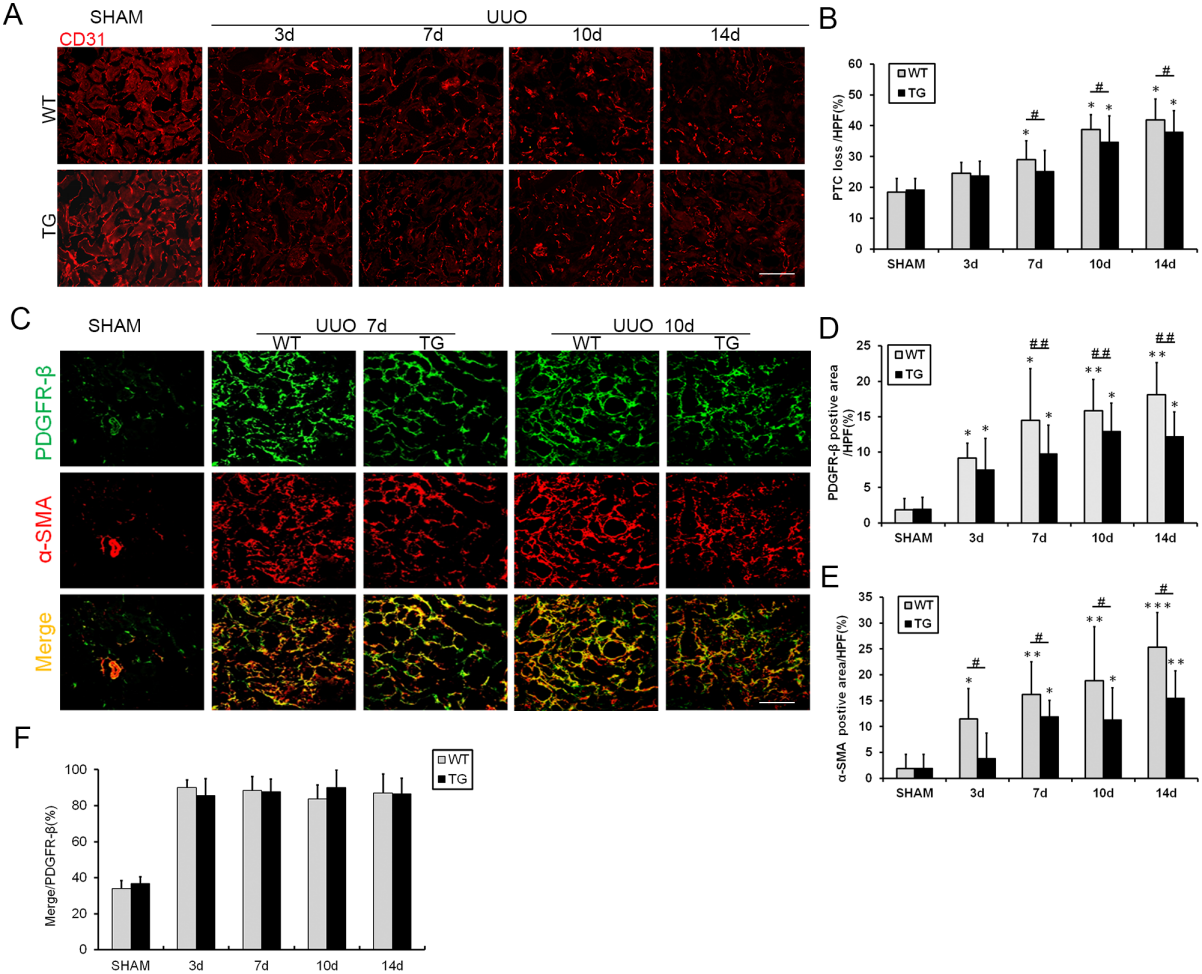
HO-1 overexpression reduced peripheral capillary loss and inhibited the activation and proliferation of renal interstitial myofibroblasts after UUO. **(A)** Representative immunofluorescence images of CD31-labeled PTCs (red) from the Sham and UUO groups on days 3, 7, 10 and 14 (magnification, ×200; bars = 250 μm). **(B)** Graph showing the morphometric quantification of CD31-positive staining. **(C)** Representative immunofluorescence images of kidney sections co-labeled with PDGFRβ (green) and α-SMA (red) in the Sham and UUO groups on days 7 and 10. PDGFRβ is a specific marker of pericytes, fibroblasts and myofibroblasts, and α-SMA labels activated fibroblasts and myofibroblasts (magnification, ×400; bars = 100 μm). **(D)** Graph displaying the morphometric quantification of the PDGFRβ-positive area per high-power field. **(E)** Histogram showing the quantification of the α-SMA -positive area per high-power field. **(F)** Graph showing the morphometric quantification ratios of the double-positive/PDGFR-β-positive areas. The data are expressed as the mean ± SD. * P<0.05, ** P<0.01, *** P<0.001 vs. WT mice of the Sham group; #P<0.05, ## P<0.01 vs. WT mice of the respective groups.

Maintenance of the microvasculature is critical for the prevention of progressive renal disease.[18, 21] Using CD31 as a marker of the endothelium, PTC density was evaluated and found to be decreased in the kidneys after UUO compared with the sham kidneys. These changes were aggravated over time with the progression of renal fibrosis. Meanwhile, the loss of PTCs was significantly improved in the TG group, especially on days 7, 10, and 14 following UUO (Fig 3A). The same result is expressed graphically in Fig 3B.

Platelet-derived growth factor receptor β (PDGFRβ), which is a common marker of fibrosis-related mesenchymal cells, including pericytes, vascular smooth muscle cells, fibroblasts, and myofibroblasts, was used to observe the proliferation of fibrosis-related cells by immunofluorescence staining. A significant increase in the PDGFRβ-positive area was observed in the interstitium of the kidneys of UUO mice compared with sham kidneys (upper panel of Fig 3C), and this increase was suppressed in the kidneys of the HO-1 transgenic mice, as shown on days 7, 10 and 14 following UUO. This result is expressed graphically in Fig 3D. Furthermore, the area that was positive for α-smooth muscle actin (α-SMA, a myofibroblast marker) was also decreased in the TG group (center panel of Fig 3C and 3E). Kidney sections were co-labeled with PDGFRβ and α-SMA to verify the composition of PDGFRβ- positive cells in renal interstitium. Nearly 90% of the cells were co-stained for PDGFRβ and α-SMA (lower panel of Fig 3C). We quantified the ratio of the double-positive area to the PDGFRβ-positive area (Fig 3F). The majority of PDGFRβ-positive cells were co-labeled with α-SMA, which is consistent with the findings of a previous study.[22]

### HO-1 overexpression regulates cellular proliferation and apoptosis after renal fibrosis induced by UUO

**Fig 4:**
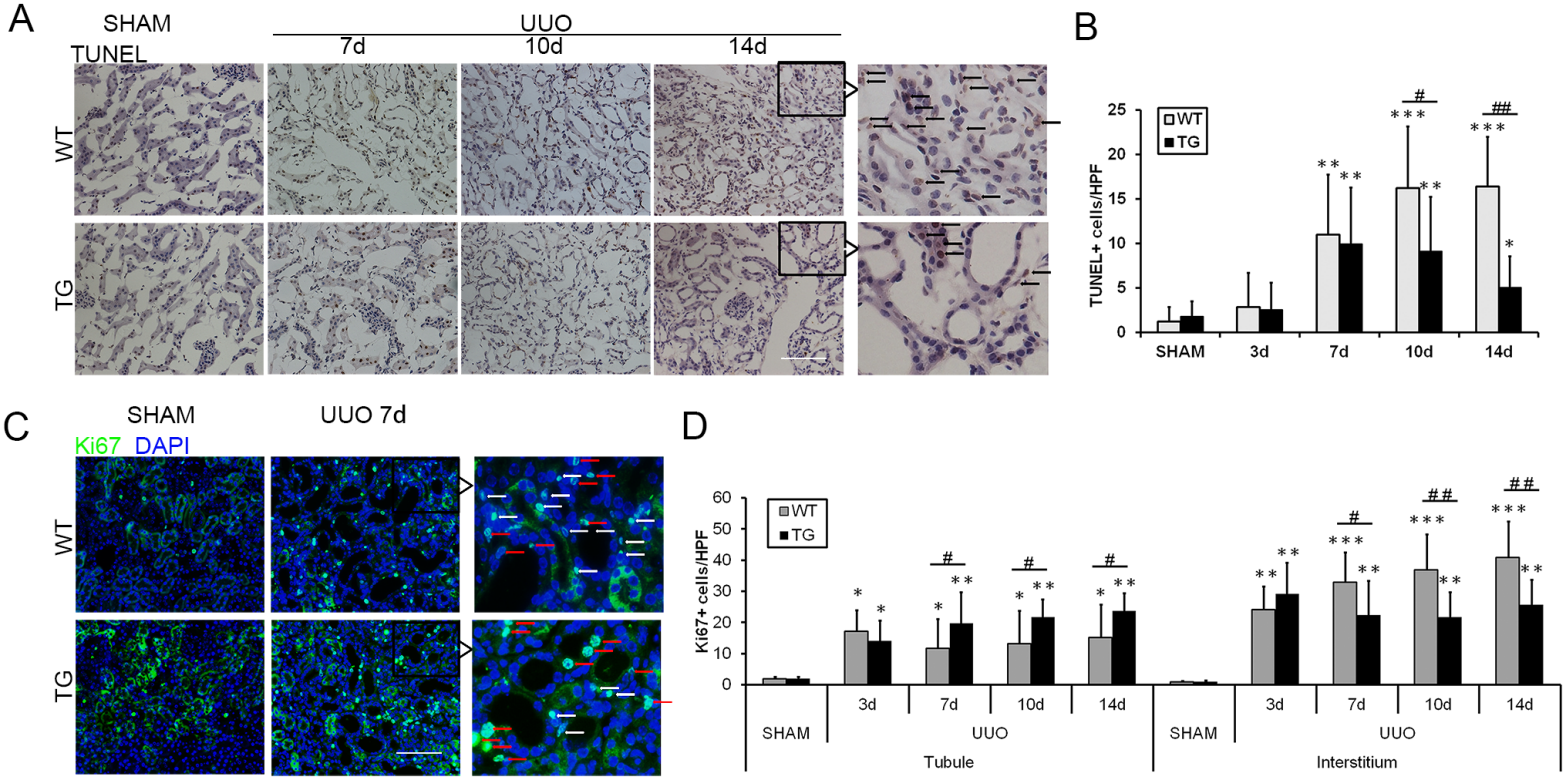
HO-1 overexpression regulated cell proliferation and apoptosis after renal fibrosis induced by UUO. **(A)** Representative images of TUNEL staining (brown) of kidney sections from the Sham and UUO groups on days 7, 10, and 14. (magnification, ×200; bars = 250 μm) **(B)** Graph indicating the number of apoptotic cells in mice after UUO. **(C)** Representative Ki67 immunofluorescence staining of kidneys with UUO at days 7 (magnification, ×200; bars = 250 μm). Red arrows indicate Ki67+ epithelial cells, and white arrows indicate Ki67+ tubulointerstitial cells. **(D)** Graph showing the number of proliferative cells in renal tubule and interstitial compartments. The data are expressed as the mean ± SD. * P<0.05, ** P<0.01, *** P<0.001 vs. WT mice of the Sham group; #P<0.05, ## P<0.01 vs. WT mice of the respective groups.

Cell cycle arrest and cellular apoptosis are two of the major epithelial mechanisms contributing to chronic tubulointerstitial fibrosis.[1, 2, 23] Therefore, apoptosis and proliferation of renal epithelial cells were assessed in our study. The number of TUNEL-positive apoptotic cells increased with the progression of renal fibrosis. HO-1 overexpression significantly reduced the number of apoptotic cells, especially on days 10 and 14 after UUO, as shown in Fig 4A and 4B. In addition, cell cycle labeling of kidney cells with the use of the pan-cell cycle marker Ki67 showed that Ki67-positive cells are not only interstitial cells but rather of tubular origin. HO-1 overexpression significantly enhanced the proliferation of epithelial cells in the kidneys of TG mice compared with those of WT mice on days 7 and 10 after UUO (Fig 4C and 4D). Concomitantly, the proliferation of tubulointerstitial cells (i.e., myofibroblasts and/or macrophages) was reduced in the TG group compared with the WT group (Fig 4C and 4D).

### HO-1 overexpression decreases tubulointerstitial inflammation in the UUO model

**Fig 5:**
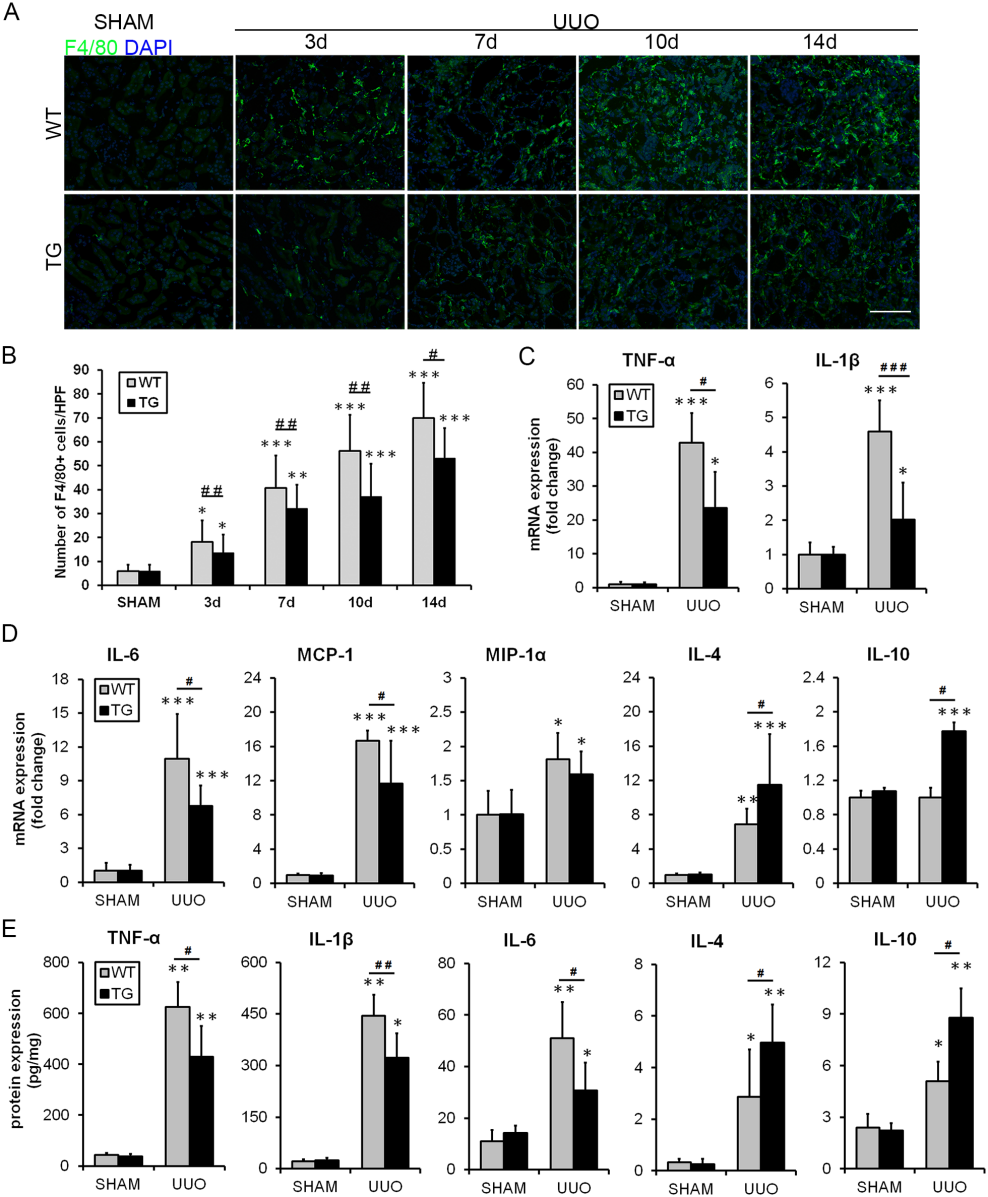
HO-1 decreased tubulointerstitial inflammation in the UUO model. **(A)** Representative immunofluorescence images of F4/80-labeled macrophages (green) from the Sham and UUO groups on days 3, 7, 10 and 14. DAPI labels the nucleus (blue). (Magnification, ×200; bars = 250 μm) **(B)** Histogram indicating the morphometric quantification of the number of F4/80-positive cells per high-power field. **(C-D)** Graph showing the mRNA expression of inflammation-related cytokines at day 7 after UUO in the TG and WT groups. **(E)** Representative ELISA for inflammatory markers (TNF-α, IL-1β, IL-6, IL-4 and IL-10) in kidney tissue homogenates of HO-1 TG mice on the 7^th^ day after UUO. The data are expressed as the mean ± SD. * P<0.05, ** P<0.01, *** P<0.001 vs. WT mice of the Sham group; #P<0.05, ## P<0.01 vs. WT mice of the respective groups.

The tubular inflammatory response and macrophage infiltration contribute to the development of renal injury and interstitial fibrosis. As depicted in Fig 5A and 5B, immunofluorescence experiments demonstrated that macrophage infiltration significantly increased in the renal interstitium after UUO, and this alteration was clearly inhibited in TG mice compared with WT mice. The mRNAs of inflammatory cytokines, such as tumor necrosis factor-α (TNF-α), interleukin (IL) -1β, IL-6, monocyte chemoattractant protein-1 (MCP-1), and macrophage inflammatory protein-1α (MIP-1α), were significantly suppressed in the kidneys of HO-1 TG mice at the 7^th^ day after UUO (representative results are shown in Fig 5C and 5D). However, the expression levels of IL-4 and IL-10 were increased in the kidneys of TG mice. Protection from macrophage infiltration was most likely caused by the decreased expression of MCP-1 and MIP-1 and the increased expression of IL-4 and IL-10 in the kidneys of HO-1 TG mice (Fig 5C and 5D). After that, the concentrations of inflammatory indices (TNF-α, IL-1β, IL-6, IL-4, IL-10) in kidney tissue homogenates of HO-1 TG mice at the 7^th^ day after UUO were detected by ELISA. The similar results were shown in Fig 5E.

### HO-1 overexpression inhibits the activation of Wnt/β-catenin signaling pathway after renal fibrosis induced by UUO

**Fig 6:**
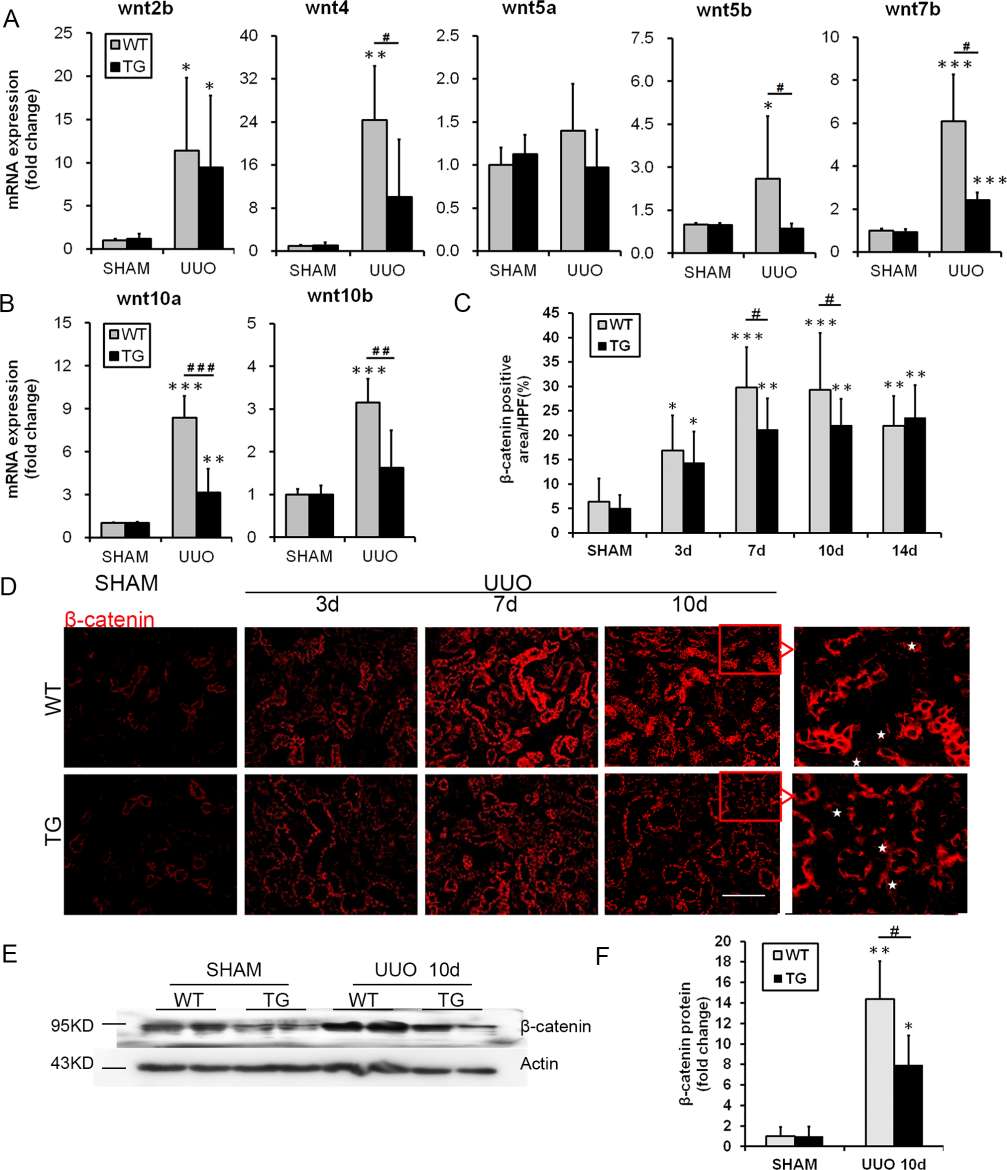
HO-1 inhibited the activation of the Wnt/ β-catenin signaling pathway after renal fibrosis induced by UUO. **(A-B)** Semi-quantitative real time-PCR of transcript levels of Wnt/β-catenin pathway ligands in the whole kidney 7 days after sham or UUO surgery. **(C)** Histogram showing the quantification of the β-catenin-positive area per high-power field. **(D)** Representative immunofluorescence images showing the expression and localization patterns of β-catenin, a common downstream mediator of the canonical Wnt signaling pathway, in the kidneys of the Sham and UUO groups at days 3, 7 and 10 (magnification, ×200; bars = 250 μm). Asterisks indicate β-catenin-positive tubulointerstitial cells. **(E-F)** Representative Western blot (E) and quantitative data (F) are presented for renal β-catenin expression 10 days after sham or UUO surgery. The data are expressed as the mean ± SD. * P<0.05, ** P<0.01, *** P<0.001 vs. WT mice of the Sham group; #P<0.05, ## P<0.01 vs. WT mice of the respective groups.

Recent findings indicate that classical Wnt/β-catenin signaling is a key contributor to renal fibrosis.[15, 16] Additionally, macrophages are a major source of functionally active Wnt ligands.[13] For this reason, the role of the Wnt/β-catenin pathway in HO-1- mediated effects needs to be clarified. In the present study, the mRNA levels of the Wnt ligands Wnt2b, Wnt4, Wnt5b, Wnt7b, Wnt10a, and Wnt10b were up-regulated in the kidneys of WT mice at the 7^th^ day after UUO compared with those of the sham mice (representative results are shown in Fig 6A and 6B). The HO-1 transgene decreased the overexpression of Wnt4, Wnt5b, Wnt7b, Wnt10a, and Wnt10b mRNAs; however, it did not significantly influence Wnt2b or Wnt5a expression. Additionally, immunostaining revealed that β-catenin expression increased after UUO induction and was predominantly localized to and increased in the renal tubular epithelium. However, β-catenin was down-regulated in the TG group compared with the WT group, especially on days 7 and 10 after UUO (Fig 6D). This result is expressed graphically in Fig 6C. Similar results were also verified by western blotting: β-catenin levels were decreased in the kidneys of TG mice compared with those of WT mice on the 10^th^ day following UUO (representative results are shown in Fig 6E and 6F).

## Discussion

Tubulointerstitial fibrosis is a common pathway among all forms of CKD and remains the best predictor of disease progression [1–3]. However, the molecular mechanisms driving fibrogenesis are still not fully understood, and few anti-fibrotic therapies are effective. Thus, the identification of better targets for future therapy is necessary. HO-1, a cytoprotective enzyme, is an emerging therapeutic molecule in a myriad of disease models.[4, 24] In the present study, we found that (1) HO-1 up-regulation in fibrotic kidneys was an important beneficial adaptation and (2) sustained overexpression of HO-1 protected tubular epithelial cells and peritubular capillaries and suppressed renal inflammation and fibrosis induced by UUO in HO-1 TG mice. The major findings of our study are that HO-1 may prevent renal fibrosis by reducing macrophage infiltration and preventing the activation of Wnt/β-catenin signaling.

HO-1 is an isoform of heme oxygenase that catalyzes the initial step of and is the rate-limiting factor in the conversion of heme to biliverdin, carbon monoxide, and iron.[4, 24] Manipulation of this enzymatic system in diverse disease models has provided substantial evidence to support its role as a cytoprotective enzyme and its adaptive up-regulation after tissue injury.[5, 7, 24] Overexpressed HO-1 was obviously observed in renal tubules and interstitium in TG mice under normal conditions. In present study, we have found that the tubules are main source of HO-1 in both WT and TG mice and the macrophages are part of origin of HO-1 after UUO injury in both groups. And it was also up-regulated in the kidneys following UUO in both TG and WT mice as a response to injury. Meanwhile, renal tubulointerstitial injury and fibrosis were reduced in the kidneys of mice with overexpressed HO-1. Our results indicated that HO-1 has a beneficial role in protection against renal fibrosis. Numerous studies have shown that bile pigments and carbon monoxide at low concentrations are cytoprotective and have a broad influence on signaling pathways, whereas iron links heme oxygenase to ferritin and other participants in iron homeostasis.[4, 24] Meanwhile, the endogenous toxicant, heme, is removed. These processes were recognized as the major mechanisms of the vasorelaxant, antioxidant, anti-inflammatory, and anti-apoptotic effects associated with organ injury, including AKI.[4] Other researchers have demonstrated additional mechanisms associated with HO-1 protection against tissue injury, which will be verified in future studies.

The histopathology of tubulointerstitial fibrosis is characterized by the deposition of ECM in association with tubular cell loss, fibroblast accumulation, infiltration of inflammatory cells, and rarefaction of the peritubular microvasculature.[1] Loss of PTCs is a characteristic feature of progressive renal disease.[25] In addition, researchers have shown that microvascular destabilization is initiated by the loss of endothelial cell (EC)-EC interactions and EC-pericyte interactions.[26, 27] Detachment of pericytes from the endothelium of PTCs under pathological conditions might be one significant cause of an unstable microvasculature and PTC rarefaction.[28] The kidneys in the present study presented obvious PTC loss after UUO, and HO-1 overexpression decreased the loss of PTC. The protection of vascular stabilization by HO-1 may be associated with an increase in circulating endothelial progenitor cells derived from the bone marrow.[29] The question of whether HO-1 is involved in maintaining EC-EC and/or EC-pericyte interactions requires further research. Meanwhile, in the current study, HO-1 overexpression inhibited the increases in PDGFRβ-positive fibrosis-related mesenchymal cells (i.e., pericytes, fibroblasts, and myofibroblasts) and α-SMA-positive myofibroblasts in the renal interstitium induced by UUO. The proliferation of tubulointerstitial cells (i.e., myofibroblasts or macrophages) was also reduced. The majority of PDGFRβ-positive cells co-stained with a myofibroblast marker, indicating that myofibroblasts constitute the majority of fibrosis-related mesenchymal cells in fibrotic kidneys. Our study demonstrated that HO-1 decreased not only the loss of PTCs but also the activation and proliferation of myofibroblasts.

Emerging evidence indicates that tubular epithelial cell necrosis, apoptosis, and growth arrest may lead to renal injury through increased fibrosis.[1, 2, 23] In the present study, we observed that the apoptosis of renal cells gradually increased with time after UUO, especially in the epithelia. HO-1 overexpression markedly inhibited this apoptosis. This anti-apoptotic effect may be mediated by the inhibition of apoptotic signaling pathways.[30] In addition, tubular cells often exhibit a tremendous capacity to regenerate, which may be crucial for recovery from renal injury.[31] The proliferation of epithelial cells was significantly enhanced in the kidneys of HO-1 TG mice after UUO. Our results suggest that HO-1 protects tubules by promoting regeneration and inhibiting the apoptosis of epithelial cells.

Inflammation contributes to tubular atrophy and interstitial fibrosis.[9] Macrophages are one of the primary cell types recruited during tubulointerstitial fibrosis.[11] The recruitment of macrophages may contribute to kidney repair at the early stages, but this repair can ultimately cause renal fibrosis.[10, 11] Regardless, ablation studies show that macrophages play important roles in stimulating fibrosis.[32] In our study, HO-1 overexpression obviously inhibited the infiltration of macrophages at every time point following UUO. Meanwhile, pro-inflammatory cytokines, such as TNF-α, IL-1β, IL-6, MCP-1, and MIP-1, which also have roles in promoting macrophage infiltration, were significantly suppressed in the kidneys of HO-1 TG mice. Furthermore, HO-1 overexpression increased the expression of IL-10, a molecule with anti-inflammatory and anti-fibrotic properties.[33, 34] Similar research had also indicated that HO-1 mediates the anti-inflammatory effect of IL-10 in mice.[35] IL-4 is also an anti-inflammatory factor,[36] and HO-1 promoted its expression after UUO. However, previous studies indicate that IL-4 can also trigger pro-fibrogenic effects by polarizing macrophages and CD4+ T lymphocytes.[37] The immunomodulatory mechanism of HO-1 thus needs to be investigated in future studies. This protection against macrophage infiltration afforded by HO-1 was at least partly due to protection against vascular endothelial damage and dysfunction, which can lead to inflammatory cell infiltration.[38] The present results demonstrate that anti-inflammatory and immunomodulatory effects are part of the mechanism by which HO-1 attenuates renal fibrosis.

Wnt/β-catenin signaling is a conserved signaling pathway that regulates cell fate, function, and phenotype during kidney development.[14] Aberrant regulation of Wnt/β-catenin has been implicated in many types of kidney diseases.[14] Activation of the canonical Wnt pathway stimulates fibroblasts and induces fibrosis,[39] including renal fibrosis.[15] Inhibiting the Wnt/β-catenin pathway attenuates renal interstitial fibrosis.[16] Other evidence has indicated that HO-1 expression can regulate the canonical Wnt signaling cascade.[40] In the current study, the Wnt ligands Wnt2b, Wnt4, Wnt5b, Wnt7b, Wnt10a, and Wnt10b, which promote the activation of the canonical Wnt pathway, were observed to be up-regulated in fibrotic kidneys. However, sustained overexpression of HO-1 selectively suppressed the up-regulation of Wnt4, Wnt5b, Wnt7b, Wnt10a, and Wnt10b. The mechanisms by which HO-1 is involved in the regulation of the Wnt pathway may be associated not only with the inhibition of some Wnt ligands but also with the inhibition of some fibrogenic signaling pathways, such as the TGF-β, CTGF, and PDGF signaling pathways. Studies have demonstrated that activation of canonical Wnt signaling is required for TGF-β-mediated fibrosis [39] and that the Wnt coreceptors, such as low-density lipoprotein receptor-related protein 6 (LRP-6), are coreceptors for multiple fibrogenic signaling pathways in myofibroblasts.[16] This proposed regulatory scheme was further verified in our study. Meanwhile, the effects of HO-1, namely, blocking activation and proliferation of established myofibroblasts and activation of TGF-β and CTGF, are partly dependent on the inhibition of downstream β-catenin, which regulates multiple fibrosis genes. Nonetheless, inhibition of HO-1 to Wnt ligands may occur via the suppression of macrophage infiltration, as macrophages are not only involved in driving chronic inflammation but also serve as a major source of functionally active Wnt ligands, including Wnt4, Wnt7b, Wnt10a, and Wnt10b.[13] Here, it is the first time to demonstrate that HO-1 suppresses the activation of Wnt/β-catenin in UUO-induced renal fibrosis. However, the conclusion that HO-1 directly regulates the Wnt/β-catenin pathway requires further investigation in the future.

In summary, our study demonstrated that HO-1, as a stress molecule, is adaptively up-regulated in UUO-induced renal damage. Moreover, sustained overexpression of HO-1 counteracts multiple detrimental renal fibrosis-associated pathological processes, such as PTC rarefaction, tubular apoptosis, and proliferation of myofibroblasts. Furthermore, our study is the first time to demonstrate that HO-1 overexpression inhibits reactivation of the Wnt/β-catenin signaling pathway, which plays crucial roles in attenuating renal fibrosis. From a practical standpoint, augmentation of the HO-1 level may be a therapeutic strategy against renal interstitial fibrosis.
